# Supplementary material for: Resolving the full spectrum of human genome variation using Linked-Reads
Source: Genome Res. 2019 Apr;29(4):635–45. doi: 10.1101/gr.234443.118 (PMC6442396; doi:10.1101/gr.234443.118)
Supplement: Supplemental Material [file supp_gr.234443.118_Supplemental_longranger-2.2.zip › longranger-2.2.tar.bz2!/lr2.2-public/lib/stan/cmdstan/stan/doc/api/html/chains_8hpp_source.html]

Stan: src/stan/mcmc/chains.hpp Source File


|  |  |
| --- | --- |
|  | Stan  2.5.0  probability, sampling & optimization |


- Main Page
- Related Pages
- Namespaces
- Classes
- Files

- File List
- File Members

All Classes Namespaces Files Functions Variables Typedefs Enumerations Enumerator Friends Macros Pages

- src
- stan
- mcmc

chains.hpp

Go to the documentation of this file.

1 #ifndef STAN\_\_MCMC\_\_CHAINS\_HPP

2 #define STAN\_\_MCMC\_\_CHAINS\_HPP

3

4 #include <algorithm>

5 #include <cmath>

6 #include <iostream>

7 #include <map>

8 #include <stdexcept>

9 #include <string>

10 #include <sstream>

11 #include <utility>

12 #include <vector>

13 #include <fstream>

14 #include <cstdlib>

15

16 #include <boost/accumulators/accumulators.hpp>

17 #include <boost/accumulators/statistics/stats.hpp>

18 #include <boost/accumulators/statistics/mean.hpp>

19 #include <boost/accumulators/statistics/tail\_quantile.hpp>

20 #include <boost/accumulators/statistics/p\_square\_quantile.hpp>

21 #include <boost/accumulators/statistics/variance.hpp>

22 #include <boost/accumulators/statistics/covariance.hpp>

23 #include <boost/accumulators/statistics/variates/covariate.hpp>

24

25 #include <boost/random/uniform\_int\_distribution.hpp>

26 #include <boost/random/additive\_combine.hpp>

27

28 #include <stan/io/stan\_csv\_reader.hpp>

29 #include <stan/math/matrix.hpp>

30 #include <stan/math/matrix/variance.hpp>

31 #include <stan/math/matrix/meta/index\_type.hpp>

32 #include <stan/prob/autocorrelation.hpp>

33 #include <stan/prob/autocovariance.hpp>

34

35

36 namespace stan {

37

38  namespace mcmc {

39

54  template <typename RNG = boost::random::ecuyer1988>

55  class chains {

56  private:

57  Eigen::Matrix<std::string, Eigen::Dynamic, 1> param\_names\_;

58  Eigen::Matrix<Eigen::MatrixXd, Eigen::Dynamic, 1> samples\_;

59  Eigen::VectorXi warmup\_;

60

61  static double mean(const Eigen::VectorXd& x) {

62  return (x.array() / x.size()).sum();

63  }

64

65  static double variance(const Eigen::VectorXd& x) {

66  double m = mean(x);

67  return ((x.array() - m) / std::sqrt((x.size() - 1.0))).square().sum();

68  }

69

70  static double sd(const Eigen::VectorXd& x) {

71  return std::sqrt(variance(x));

72  }

73

74

75  static double covariance(const Eigen::VectorXd& x,

76  const Eigen::VectorXd& y) {

77  if (x.rows() != y.rows())

78  std::cerr << "warning: covariance of different length chains";

79  using boost::accumulators::accumulator\_set;

80  using boost::accumulators::stats;

81  using boost::accumulators::tag::variance;

82  using boost::accumulators::tag::covariance;

83  using boost::accumulators::tag::covariate1;

84

85  accumulator\_set<double, stats<covariance<double, covariate1> > > acc;

86

87  int M = std::min(x.size(), y.size());

88  for (int i = 0; i < M; i++)

89  acc(x(i), boost::accumulators::covariate1=y(i));

90

91  return boost::accumulators::covariance(acc) \* M / (M-1);

92  }

93

94  static double correlation(const Eigen::VectorXd& x,

95  const Eigen::VectorXd& y) {

96  if (x.rows() != y.rows())

97  std::cerr << "warning: covariance of different length chains";

98  using boost::accumulators::accumulator\_set;

99  using boost::accumulators::stats;

100  using boost::accumulators::tag::variance;

101  using boost::accumulators::tag::covariance;

102  using boost::accumulators::tag::covariate1;

103

104  accumulator\_set<double, stats<variance,

105  covariance<double, covariate1> > > acc\_xy;

106  accumulator\_set<double, stats<variance> > acc\_y;

107

108  int M = std::min(x.size(), y.size());

109  for (int i = 0; i < M; i++) {

110  acc\_xy(x(i), boost::accumulators::covariate1=y(i));

111  acc\_y(y(i));

112  }

113

114  double cov = boost::accumulators::covariance(acc\_xy);

115  if (cov > -1e-8 && cov < 1e-8)

116  return cov;

117  return cov / std::sqrt(boost::accumulators::variance(acc\_xy)

118  \* boost::accumulators::variance(acc\_y));

119  }

120

121  static double quantile(const Eigen::VectorXd& x, const double prob) {

122  using boost::accumulators::accumulator\_set;

123  using boost::accumulators::left;

124  using boost::accumulators::quantile\_probability;

125  using boost::accumulators::right;

126  using boost::accumulators::stats;

127  using boost::accumulators::tag::tail;

128  using boost::accumulators::tag::tail\_quantile;

129  double M = x.rows();

130  //size\_t cache\_size = std::min(prob, 1-prob)\*M + 2;

131  size\_t cache\_size = M;

132

133  if (prob < 0.5) {

134  accumulator\_set<double, stats<tail\_quantile<left> > >

135  acc(tail<left>::cache\_size = cache\_size);

136  for (int i = 0; i < M; i++)

137  acc(x(i));

138  return boost::accumulators::quantile(acc, quantile\_probability=prob);

139  }

140  accumulator\_set<double, stats<tail\_quantile<right> > >

141  acc(tail<right>::cache\_size = cache\_size);

142  for (int i = 0; i < M; i++)

143  acc(x(i));

144  return boost::accumulators::quantile(acc, quantile\_probability=prob);

145  }

146

147  static Eigen::VectorXd

148  quantiles(const Eigen::VectorXd& x, const Eigen::VectorXd& probs)

149  {

150  using boost::accumulators::accumulator\_set;

151  using boost::accumulators::left;

152  using boost::accumulators::quantile\_probability;

153  using boost::accumulators::right;

154  using boost::accumulators::stats;

155  using boost::accumulators::tag::tail;

156  using boost::accumulators::tag::tail\_quantile;

157  double M = x.rows();

158

159  //size\_t cache\_size = M/2 + 2;

160  size\_t cache\_size = M;

161

162  accumulator\_set<double, stats<tail\_quantile<left> > >

163  acc\_left(tail<left>::cache\_size = cache\_size);

164  accumulator\_set<double, stats<tail\_quantile<right> > >

165  acc\_right(tail<right>::cache\_size = cache\_size);

166

167  for (int i = 0; i < M; i++) {

168  acc\_left(x(i));

169  acc\_right(x(i));

170  }

171

172  Eigen::VectorXd q(probs.size());

173  for (int i = 0; i < probs.size(); i++) {

174  if (probs(i) < 0.5)

175  q(i) = boost::accumulators::quantile(acc\_left,

176  quantile\_probability=probs(i));

177  else

178  q(i) = boost::accumulators::quantile(acc\_right,

179  quantile\_probability=probs(i));

180  }

181  return q;

182  }

183

184  static Eigen::VectorXd autocorrelation(const Eigen::VectorXd& x) {

185  using std::vector;

186  using stan::math::index\_type;

187  typedef typename index\_type<vector<double> >::type idx\_t;

188

189  std::vector<double> ac;

190  std::vector<double> sample(x.size());

191  for (int i = 0; i < x.size(); i++)

192  sample[i] = x(i);

193  stan::prob::autocorrelation(sample, ac);

194

195  Eigen::VectorXd ac2(ac.size());

196  for (idx\_t i = 0; i < ac.size(); i++)

197  ac2(i) = ac[i];

198  return ac2;

199  }

200

201  static Eigen::VectorXd autocovariance(const Eigen::VectorXd& x) {

202  using std::vector;

203  using stan::math::index\_type;

204  typedef typename index\_type<vector<double> >::type idx\_t;

205

206  std::vector<double> ac;

207  std::vector<double> sample(x.size());

208  for (int i = 0; i < x.size(); i++)

209  sample[i] = x(i);

210  stan::prob::autocovariance(sample, ac);

211

212  Eigen::VectorXd ac2(ac.size());

213  for (idx\_t i = 0; i < ac.size(); i++)

214  ac2(i) = ac[i];

215  return ac2;

216  }

217

233  double effective\_sample\_size(const Eigen::Matrix<Eigen::VectorXd,

234  Eigen::Dynamic, 1> &samples) const {

235  int chains = samples.size();

236

237  // need to generalize to each jagged samples per chain

238  int n\_samples = samples(0).size();

239  for (int chain = 1; chain < chains; chain++) {

240  n\_samples = std::min(n\_samples, int(samples(chain).size()));

241  }

242

243  Eigen::Matrix<Eigen::VectorXd, Eigen::Dynamic, 1> acov(chains);

244  for (int chain = 0; chain < chains; chain++) {

245  acov(chain) = autocovariance(samples(chain));

246  }

247

248  Eigen::VectorXd chain\_mean(chains);

249  Eigen::VectorXd chain\_var(chains);

250  for (int chain = 0; chain < chains; chain++) {

251  double n\_kept\_samples = num\_kept\_samples(chain);

252  chain\_mean(chain) = mean(samples(chain));

253  chain\_var(chain) = acov(chain)(0)\*n\_kept\_samples/(n\_kept\_samples-1);

254  }

255

256  double mean\_var = mean(chain\_var);

257  double var\_plus = mean\_var\*(n\_samples-1)/n\_samples;

258  if (chains > 1)

259  var\_plus += variance(chain\_mean);

260  Eigen::VectorXd rho\_hat\_t(n\_samples);

261  rho\_hat\_t.setZero();

262  double rho\_hat = 0;

263  int max\_t = 0;

264  for (int t = 1; (t < n\_samples && rho\_hat >= 0); t++) {

265  Eigen::VectorXd acov\_t(chains);

266  for (int chain = 0; chain < chains; chain++) {

267  acov\_t(chain) = acov(chain)(t);

268  }

269  rho\_hat = 1 - (mean\_var - mean(acov\_t)) / var\_plus;

270  if (rho\_hat >= 0)

271  rho\_hat\_t(t) = rho\_hat;

272  max\_t = t;

273  }

274  double ess = chains \* n\_samples;

275  if (max\_t > 1) {

276  ess /= 1 + 2 \* rho\_hat\_t.sum();

277  }

278  return ess;

279  }

280

294  double

295  split\_potential\_scale\_reduction(

296  const Eigen::Matrix<Eigen::VectorXd,

297  Eigen::Dynamic, 1> &samples) const {

298  int chains = samples.size();

299  int n\_samples = samples(0).size();

300  for (int chain = 1; chain < chains; chain++) {

301  n\_samples = std::min(n\_samples, int(samples(chain).size()));

302  }

303  if (n\_samples % 2 == 1)

304  n\_samples--;

305  int n = n\_samples / 2;

306

307  Eigen::VectorXd split\_chain\_mean(2\*chains);

308  Eigen::VectorXd split\_chain\_var(2\*chains);

309

310  for (int chain = 0; chain < chains; chain++) {

311  split\_chain\_mean(2\*chain) = mean(samples(chain).topRows(n));

312  split\_chain\_mean(2\*chain+1) = mean(samples(chain).bottomRows(n));

313

314  split\_chain\_var(2\*chain) = variance(samples(chain).topRows(n));

315  split\_chain\_var(2\*chain+1) = variance(samples(chain).bottomRows(n));

316  }

317

318  double var\_between = n \* variance(split\_chain\_mean);

319  double var\_within = mean(split\_chain\_var);

320

321  // rewrote [(n-1)\*W/n + B/n]/W as (n-1+ B/W)/n

322  return sqrt((var\_between/var\_within + n-1)/n);

323  }

324

325  public:

326  chains(const Eigen::Matrix<std::string, Eigen::Dynamic, 1>& param\_names)

327  : param\_names\_(param\_names) { }

328

329  chains(const std::vector<std::string>& param\_names) : param\_names\_(param\_names.size()) {

330  for (size\_t i = 0; i < param\_names.size(); i++)

331  param\_names\_(i) = param\_names[i];

332  }

333

334  chains(const stan::io::stan\_csv& stan\_csv)

335  : param\_names\_(stan\_csv.header) {

336  if (stan\_csv.samples.rows() > 0)

337  add(stan\_csv);

338  }

339

340  inline int num\_chains() const {

341  return samples\_.size();

342  }

343

344  inline int num\_params() const {

345  return param\_names\_.size();

346  }

347

348  const Eigen::Matrix<std::string, Eigen::Dynamic, 1>& param\_names() const {

349  return param\_names\_;

350  }

351

352  const std::string& param\_name(int j) const {

353  return param\_names\_(j);

354  }

355

356  int index(const std::string& name) const {

357  int index = -1;

358  for (int i = 0; i < param\_names\_.size(); i++)

359  if (param\_names\_(i) == name)

360  return i;

361  return index;

362  }

363

364  void set\_warmup(const int chain, const int warmup) {

365  warmup\_(chain) = warmup;

366  }

367

368  void set\_warmup(const int warmup) {

369  warmup\_.setConstant(warmup);

370  }

371

372  const Eigen::VectorXi& warmup() const {

373  return warmup\_;

374  }

375

376  int warmup(const int chain) const {

377  return warmup\_(chain);

378  }

379

380  int num\_samples(const int chain) const {

381  return samples\_(chain).rows();

382  }

383

384  int num\_samples() const {

385  int n = 0;

386  for (int chain = 0; chain < num\_chains(); chain++)

387  n += num\_samples(chain);

388  return n;

389  }

390

391  int num\_kept\_samples(const int chain) const {

392  return num\_samples(chain) - warmup(chain);

393  }

394

395  int num\_kept\_samples() const {

396  int n = 0;

397  for (int chain = 0; chain < num\_chains(); chain++)

398  n += num\_kept\_samples(chain);

399  return n;

400  }

401

402  void add(const int chain,

403  const Eigen::MatrixXd& sample) {

404  if (sample.cols() != num\_params())

405  throw std::invalid\_argument("add(chain,sample): number of columns"

406  " in sample does not match chains");

407  if (num\_chains() == 0 || chain >= num\_chains()) {

408  int n = num\_chains();

409

410  // Need this block for Windows. conservativeResize

411  // does not keep the references.

412  Eigen::Matrix<Eigen::MatrixXd, Eigen::Dynamic, 1>

413  samples\_copy(num\_chains());

414  Eigen::VectorXi warmup\_copy(num\_chains());

415  for (int i = 0; i < n; i++) {

416  samples\_copy(i) = samples\_(i);

417  warmup\_copy(i) = warmup\_(i);

418  }

419

420  samples\_.resize(chain+1);

421  warmup\_.resize(chain+1);

422  for (int i = 0; i < n; i++) {

423  samples\_(i) = samples\_copy(i);

424  warmup\_(i) = warmup\_copy(i);

425  }

426  for (int i = n; i < chain+1; i++) {

427  samples\_(i) = Eigen::MatrixXd(0, num\_params());

428  warmup\_(i) = 0;

429  }

430  }

431  int row = samples\_(chain).rows();

432  Eigen::MatrixXd new\_samples(row+sample.rows(), num\_params());

433  new\_samples << samples\_(chain), sample;

434  samples\_(chain) = new\_samples;

435  }

436

437  void add(const Eigen::MatrixXd& sample) {

438  if (sample.rows() == 0)

439  return;

440  if (sample.cols() != num\_params())

441  throw std::invalid\_argument("add(sample): number of columns in"

442  " sample does not match chains");

443  add(num\_chains(), sample);

444  }

445

453  void add(const std::vector<std::vector<double> >& sample) {

454  int n\_row = sample.size();

455  if (n\_row == 0)

456  return;

457  int n\_col = sample[0].size();

458  Eigen::MatrixXd sample\_copy(n\_row, n\_col);

459  for (int i = 0; i < n\_row; i++) {

460  sample\_copy.row(i)

461  = Eigen::VectorXd::Map(&sample[i][0], sample[0].size());

462  }

463  add(sample\_copy);

464  }

465

466  void add(const stan::io::stan\_csv& stan\_csv) {

467  if (stan\_csv.header.size() != num\_params())

468  throw std::invalid\_argument("add(stan\_csv): number of columns in"

469  " sample does not match chains");

470  if (!param\_names\_.cwiseEqual(stan\_csv.header).all()) {

471  throw std::invalid\_argument("add(stan\_csv): header does not match"

472  " chain's header");

473  }

474  add(stan\_csv.samples);

475  if (stan\_csv.metadata.save\_warmup)

476  set\_warmup(num\_chains()-1, stan\_csv.metadata.num\_warmup);

477  }

478

479  Eigen::VectorXd samples(const int chain, const int index) const {

480  return samples\_(chain).col(index).bottomRows(num\_kept\_samples(chain));

481  }

482

483  Eigen::VectorXd samples(const int index) const {

484  Eigen::VectorXd s(num\_kept\_samples());

485  int start = 0;

486  for (int chain = 0; chain < num\_chains(); chain++) {

487  int n = num\_kept\_samples(chain);

488  s.middleRows(start,n) = samples\_(chain).col(index).bottomRows(n);

489  start += n;

490  }

491  return s;

492  }

493

494  Eigen::VectorXd samples(const int chain, const std::string& name) const {

495  return samples(chain,index(name));

496  }

497

498  Eigen::VectorXd samples(const std::string& name) const {

499  return samples(index(name));

500  }

501

502  double mean(const int chain, const int index) const {

503  return mean(samples(chain,index));

504  }

505

506  double mean(const int index) const {

507  return mean(samples(index));

508  }

509

510  double mean(const int chain, const std::string& name) const {

511  return mean(chain, index(name));

512  }

513

514  double mean(const std::string& name) const {

515  return mean(index(name));

516  }

517

518  double sd(const int chain, const int index) const {

519  return sd(samples(chain,index));

520  }

521

522  double sd(const int index) const {

523  return sd(samples(index));

524  }

525

526  double sd(const int chain, const std::string& name) const {

527  return sd(chain, index(name));

528  }

529

530  double sd(const std::string& name) const {

531  return sd(index(name));

532  }

533

534  double variance(const int chain, const int index) const {

535  return variance(samples(chain,index));

536  }

537

538  double variance(const int index) const {

539  return variance(samples(index));

540  }

541

542  double variance(const int chain, const std::string& name) const {

543  return variance(chain, index(name));

544  }

545

546  double variance(const std::string& name) const {

547  return variance(index(name));

548  }

549

550  double

551  covariance(const int chain, const int index1, const int index2) const {

552  return covariance(samples(chain,index1), samples(chain,index2));

553  }

554

555  double covariance(const int index1, const int index2) const {

556  return covariance(samples(index1), samples(index2));

557  }

558

559  double covariance(const int chain, const std::string& name1,

560  const std::string& name2) const {

561  return covariance(chain, index(name1), index(name2));

562  }

563

564  double

565  covariance(const std::string& name1, const std::string& name2) const {

566  return covariance(index(name1), index(name2));

567  }

568

569  double

570  correlation(const int chain, const int index1, const int index2) const {

571  return correlation(samples(chain,index1),samples(chain,index2));

572  }

573

574  double correlation(const int index1, const int index2) const {

575  return correlation(samples(index1),samples(index2));

576  }

577

578  double correlation(const int chain, const std::string& name1,

579  const std::string& name2) const {

580  return correlation(chain, index(name1), index(name2));

581  }

582

583  double

584  correlation(const std::string& name1, const std::string& name2) const {

585  return correlation(index(name1), index(name2));

586  }

587

588  double

589  quantile(const int chain, const int index, const double prob) const {

590  return quantile(samples(chain,index), prob);

591  }

592

593  double quantile(const int index, const double prob) const {

594  return quantile(samples(index), prob);

595  }

596

597  double quantile(int chain, const std::string& name, double prob) const {

598  return quantile(chain, index(name), prob);

599  }

600

601  double quantile(const std::string& name, const double prob) const {

602  return quantile(index(name), prob);

603  }

604

605  Eigen::VectorXd

606  quantiles(int chain, int index, const Eigen::VectorXd& probs) const {

607  return quantiles(samples(chain,index), probs);

608  }

609

610  Eigen::VectorXd quantiles(int index, const Eigen::VectorXd& probs) const {

611  return quantiles(samples(index), probs);

612  }

613

614  Eigen::VectorXd

615  quantiles(int chain, const std::string& name,

616  const Eigen::VectorXd& probs) const {

617  return quantiles(chain, index(name), probs);

618  }

619

620  Eigen::VectorXd

621  quantiles(const std::string& name, const Eigen::VectorXd& probs) const {

622  return quantiles(index(name), probs);

623  }

624

625  Eigen::Vector2d central\_interval(int chain, int index,

626  double prob) const {

627  double low\_prob = (1-prob)/2;

628  double high\_prob = 1-low\_prob;

629

630  Eigen::Vector2d interval;

631  interval

632  << quantile(chain,index,low\_prob), quantile(chain,index,high\_prob);

633  return interval;

634  }

635

636  Eigen::Vector2d central\_interval(int index, double prob) const {

637  double low\_prob = (1-prob)/2;

638  double high\_prob = 1-low\_prob;

639

640  Eigen::Vector2d interval;

641  interval << quantile(index,low\_prob), quantile(index,high\_prob);

642  return interval;

643  }

644

645  Eigen::Vector2d

646  central\_interval(int chain, const std::string& name,

647  double prob) const {

648  return central\_interval(chain, index(name), prob);

649  }

650

651  Eigen::Vector2d central\_interval(const std::string& name,

652  double prob) const {

653  return central\_interval(index(name), prob);

654  }

655

656  Eigen::VectorXd autocorrelation(const int chain, const int index) const {

657  return autocorrelation(samples(chain, index));

658  }

659

660  Eigen::VectorXd autocorrelation(int chain,

661  const std::string& name) const {

662  return autocorrelation(chain, index(name));

663  }

664

665  Eigen::VectorXd autocovariance(const int chain, const int index) const {

666  return autocovariance(samples(chain,index));

667  }

668

669  Eigen::VectorXd autocovariance(int chain, const std::string& name) const {

670  return autocovariance(chain, index(name));

671  }

672

673  // FIXME: reimplement using autocorrelation.

674  double effective\_sample\_size(const int index) const {

675  Eigen::Matrix<Eigen::VectorXd, Eigen::Dynamic, 1>

676  samples(num\_chains());

677  for (int chain = 0; chain < num\_chains(); chain++) {

678  samples(chain) = this->samples(chain, index);

679  }

680  return effective\_sample\_size(samples);

681  }

682

683  double effective\_sample\_size(const std::string& name) const {

684  return effective\_sample\_size(index(name));

685  }

686

687  double split\_potential\_scale\_reduction(const int index) const

688  {

689  Eigen::Matrix<Eigen::VectorXd, Eigen::Dynamic, 1>

690  samples(num\_chains());

691  for (int chain = 0; chain < num\_chains(); chain++) {

692  samples(chain) = this->samples(chain, index);

693  }

694  return split\_potential\_scale\_reduction(samples);

695  }

696

697  double split\_potential\_scale\_reduction(const std::string& name) const {

698  return split\_potential\_scale\_reduction(index(name));

699  }

700  };

701

702  }

703 }

704

705 #endif

stan::mcmc::chains::warmup

int warmup(const int chain) const

**Definition:** chains.hpp:376

stan::mcmc::chains::num\_kept\_samples

int num\_kept\_samples() const

**Definition:** chains.hpp:395

stan::mcmc::chains::sd

double sd(const std::string &name) const

**Definition:** chains.hpp:530

stan::mcmc::chains::set\_warmup

void set\_warmup(const int warmup)

**Definition:** chains.hpp:368

stan::mcmc::chains::samples

Eigen::VectorXd samples(const int chain, const std::string &name) const

**Definition:** chains.hpp:494

stan::mcmc::chains::covariance

double covariance(const std::string &name1, const std::string &name2) const

**Definition:** chains.hpp:565

stan::mcmc::chains::param\_name

const std::string & param\_name(int j) const

**Definition:** chains.hpp:352

stan::mcmc::chains::correlation

double correlation(const int index1, const int index2) const

**Definition:** chains.hpp:574

stan::io::stan\_csv::samples

Eigen::MatrixXd samples

**Definition:** stan\_csv\_reader.hpp:64

stan::mcmc::chains::quantile

double quantile(const std::string &name, const double prob) const

**Definition:** chains.hpp:601

stan::mcmc::chains::mean

double mean(const int chain, const int index) const

**Definition:** chains.hpp:502

stan::mcmc::chains::quantiles

Eigen::VectorXd quantiles(int chain, const std::string &name, const Eigen::VectorXd &probs) const

**Definition:** chains.hpp:615

stan::mcmc::chains::quantile

double quantile(const int index, const double prob) const

**Definition:** chains.hpp:593

stan::math::row

Eigen::Matrix< T, 1, Eigen::Dynamic > row(const Eigen::Matrix< T, Eigen::Dynamic, Eigen::Dynamic > &m, size\_t i)

Return the specified row of the specified matrix, using start-at-1 indexing.

**Definition:** row.hpp:26

stan::mcmc::chains::chains

chains(const stan::io::stan\_csv &stan\_csv)

**Definition:** chains.hpp:334

stan::mcmc::chains::add

void add(const stan::io::stan\_csv &stan\_csv)

**Definition:** chains.hpp:466

stan::mcmc::chains::autocovariance

Eigen::VectorXd autocovariance(int chain, const std::string &name) const

**Definition:** chains.hpp:669

stan::mcmc::chains::samples

Eigen::VectorXd samples(const int chain, const int index) const

**Definition:** chains.hpp:479

stan::mcmc::chains::sd

double sd(const int chain, const int index) const

**Definition:** chains.hpp:518

stan::mcmc::chains::index

int index(const std::string &name) const

**Definition:** chains.hpp:356

autocorrelation.hpp

stan::mcmc::chains::central\_interval

Eigen::Vector2d central\_interval(int index, double prob) const

**Definition:** chains.hpp:636

stan::mcmc::chains::correlation

double correlation(const std::string &name1, const std::string &name2) const

**Definition:** chains.hpp:584

stan\_csv\_reader.hpp

stan::mcmc::chains::mean

double mean(const std::string &name) const

**Definition:** chains.hpp:514

autocovariance.hpp

variance.hpp

stan::mcmc::chains::variance

double variance(const std::string &name) const

**Definition:** chains.hpp:546

stan::mcmc::chains::sd

double sd(const int index) const

**Definition:** chains.hpp:522

stan::agrad::sum

fvar< T > sum(const Eigen::Matrix< fvar< T >, R, C > &m)

**Definition:** sum.hpp:14

stan::mcmc::chains::split\_potential\_scale\_reduction

double split\_potential\_scale\_reduction(const int index) const

**Definition:** chains.hpp:687

stan::mcmc::chains::variance

double variance(const int chain, const int index) const

**Definition:** chains.hpp:534

stan::mcmc::chains::quantile

double quantile(int chain, const std::string &name, double prob) const

**Definition:** chains.hpp:597

stan::mcmc::chains::covariance

double covariance(const int chain, const int index1, const int index2) const

**Definition:** chains.hpp:551

stan::mcmc::chains::effective\_sample\_size

double effective\_sample\_size(const int index) const

**Definition:** chains.hpp:674

stan::math::index\_type

Primary template class for the metaprogram to compute the index type of a container.

**Definition:** index\_type.hpp:21

stan::mcmc::chains::correlation

double correlation(const int chain, const int index1, const int index2) const

**Definition:** chains.hpp:570

stan::mcmc::chains::mean

double mean(const int chain, const std::string &name) const

**Definition:** chains.hpp:510

stan::mcmc::chains::sd

double sd(const int chain, const std::string &name) const

**Definition:** chains.hpp:526

stan::prob::autocovariance

void autocovariance(const std::vector< T > &y, std::vector< T > &acov, Eigen::FFT< T > &fft)

Write autocovariance estimates for every lag for the specified input sequence into the specified resu...

**Definition:** autocovariance.hpp:33

stan::mcmc::chains::covariance

double covariance(const int index1, const int index2) const

**Definition:** chains.hpp:555

stan::mcmc::chains::add

void add(const std::vector< std::vector< double > > &sample)

Convert a vector of vector<double> to Eigen::MatrixXd.

**Definition:** chains.hpp:453

stan::mcmc::chains::param\_names

const Eigen::Matrix< std::string, Eigen::Dynamic, 1 > & param\_names() const

**Definition:** chains.hpp:348

stan::mcmc::chains::autocorrelation

Eigen::VectorXd autocorrelation(const int chain, const int index) const

**Definition:** chains.hpp:656

stan::agrad::sqrt

fvar< T > sqrt(const fvar< T > &x)

**Definition:** sqrt.hpp:15

stan::mcmc::chains::variance

double variance(const int chain, const std::string &name) const

**Definition:** chains.hpp:542

stan::mcmc::chains::chains

chains(const std::vector< std::string > &param\_names)

**Definition:** chains.hpp:329

stan::mcmc::chains::samples

Eigen::VectorXd samples(const int index) const

**Definition:** chains.hpp:483

stan::io::stan\_csv

**Definition:** stan\_csv\_reader.hpp:60

stan::math::tail

Eigen::Matrix< T, Eigen::Dynamic, 1 > tail(const Eigen::Matrix< T, Eigen::Dynamic, 1 > &v, size\_t n)

Return the specified number of elements as a vector from the back of the specified vector...

**Definition:** tail.hpp:22

index\_type.hpp

stan::mcmc::chains::num\_samples

int num\_samples(const int chain) const

**Definition:** chains.hpp:380

stan::mcmc::chains::correlation

double correlation(const int chain, const std::string &name1, const std::string &name2) const

**Definition:** chains.hpp:578

stan::mcmc::chains::add

void add(const Eigen::MatrixXd &sample)

**Definition:** chains.hpp:437

stan::mcmc::chains::effective\_sample\_size

double effective\_sample\_size(const std::string &name) const

**Definition:** chains.hpp:683

stan::mcmc::chains::variance

double variance(const int index) const

**Definition:** chains.hpp:538

stan::mcmc::chains::mean

double mean(const int index) const

**Definition:** chains.hpp:506

stan::io::stan\_csv\_metadata::num\_warmup

size\_t num\_warmup

**Definition:** stan\_csv\_reader.hpp:28

stan::io::stan\_csv\_metadata::save\_warmup

bool save\_warmup

**Definition:** stan\_csv\_reader.hpp:29

stan::mcmc::chains

An mcmc::chains object stores parameter names and dimensionalities along with samples from multiple c...

**Definition:** chains.hpp:55

stan::mcmc::chains::num\_params

int num\_params() const

**Definition:** chains.hpp:344

stan::mcmc::chains::central\_interval

Eigen::Vector2d central\_interval(int chain, const std::string &name, double prob) const

**Definition:** chains.hpp:646

stan::mcmc::chains::autocovariance

Eigen::VectorXd autocovariance(const int chain, const int index) const

**Definition:** chains.hpp:665

stan::mcmc::chains::split\_potential\_scale\_reduction

double split\_potential\_scale\_reduction(const std::string &name) const

**Definition:** chains.hpp:697

stan::math::e

double e()

Return the base of the natural logarithm.

**Definition:** constants.hpp:86

stan::mcmc::chains::add

void add(const int chain, const Eigen::MatrixXd &sample)

**Definition:** chains.hpp:402

stan::mcmc::chains::central\_interval

Eigen::Vector2d central\_interval(const std::string &name, double prob) const

**Definition:** chains.hpp:651

stan::mcmc::chains::quantiles

Eigen::VectorXd quantiles(int index, const Eigen::VectorXd &probs) const

**Definition:** chains.hpp:610

stan::math::size

int size(const std::vector< T > &x)

**Definition:** size.hpp:11

stan::mcmc::chains::autocorrelation

Eigen::VectorXd autocorrelation(int chain, const std::string &name) const

**Definition:** chains.hpp:660

stan::mcmc::chains::num\_chains

int num\_chains() const

**Definition:** chains.hpp:340

stan::mcmc::chains::num\_samples

int num\_samples() const

**Definition:** chains.hpp:384

stan::mcmc::chains::quantiles

Eigen::VectorXd quantiles(int chain, int index, const Eigen::VectorXd &probs) const

**Definition:** chains.hpp:606

stan::math::min

double min(const double a, const double b)

**Definition:** min.hpp:7

stan::mcmc::chains::chains

chains(const Eigen::Matrix< std::string, Eigen::Dynamic, 1 > &param\_names)

**Definition:** chains.hpp:326

stan::agrad::variance

var variance(const std::vector< var > &v)

Return the sample variance of the specified standard vector.

**Definition:** variance.hpp:51

stan::mcmc::chains::quantiles

Eigen::VectorXd quantiles(const std::string &name, const Eigen::VectorXd &probs) const

**Definition:** chains.hpp:621

stan::io::stan\_csv::metadata

stan\_csv\_metadata metadata

**Definition:** stan\_csv\_reader.hpp:61

stan::mcmc::chains::covariance

double covariance(const int chain, const std::string &name1, const std::string &name2) const

**Definition:** chains.hpp:559

stan::mcmc::sample

**Definition:** sample.hpp:13

stan::mcmc::chains::quantile

double quantile(const int chain, const int index, const double prob) const

**Definition:** chains.hpp:589

stan::io::stan\_csv::header

Eigen::Matrix< std::string, Eigen::Dynamic, 1 > header

**Definition:** stan\_csv\_reader.hpp:62

stan::common::sample

void sample(stan::mcmc::base\_mcmc \*sampler, int num\_warmup, int num\_samples, int num\_thin, int refresh, bool save, stan::io::mcmc\_writer< Model, SampleRecorder, DiagnosticRecorder, MessageRecorder > &writer, stan::mcmc::sample &init\_s, Model &model, RNG &base\_rng, const std::string &prefix, const std::string &suffix, std::ostream &o, StartTransitionCallback &callback)

**Definition:** sample.hpp:13

stan::mcmc::chains::samples

Eigen::VectorXd samples(const std::string &name) const

**Definition:** chains.hpp:498

stan::mcmc::chains::central\_interval

Eigen::Vector2d central\_interval(int chain, int index, double prob) const

**Definition:** chains.hpp:625

stan::mcmc::chains::warmup

const Eigen::VectorXi & warmup() const

**Definition:** chains.hpp:372

stan::prob::autocorrelation

void autocorrelation(const std::vector< T > &y, std::vector< T > &ac, Eigen::FFT< T > &fft)

Write autocorrelation estimates for every lag for the specified input sequence into the specified res...

**Definition:** autocorrelation.hpp:56

stan::mcmc::chains::num\_kept\_samples

int num\_kept\_samples(const int chain) const

**Definition:** chains.hpp:391

stan::mcmc::chains::set\_warmup

void set\_warmup(const int chain, const int warmup)

**Definition:** chains.hpp:364

matrix.hpp


---

[ Stan Home Page ]

*© 2011–2014,
Stan Development Team.*
